# The Hedgehog Signaling Pathway: A Viable Target in Breast Cancer?

**DOI:** 10.3390/cancers11081126

**Published:** 2019-08-07

**Authors:** Priyanka Bhateja, Mathew Cherian, Sarmila Majumder, Bhuvaneswari Ramaswamy

**Affiliations:** Division of Medical Oncology, Department of Internal medicine, James Comprehensive Cancer Center, Ohio State University, Columbus, OH 43210, USA

**Keywords:** hedgehog, GLI1, breast cancer

## Abstract

The hedgehog (Hh) pathway plays a key role in embryonic development and stem cell programs. Deregulation of the Hh pathway is a key driver of basal cell carcinoma, and therapeutic targeting led to approval of Hh inhibitor, vismodegib, in the management of this cancer. The Hh pathway is implicated in other malignancies including hormone receptor (HR+) positive and triple negative breast cancer (TNBC). Hh signaling, which is activated in human mammary stem cells, results in activation of glioma-associated oncogene (GLI) transcription factors. High GLI1 expression correlates with worse outcomes in breast cancer. Non-canonical GLI1 activation is one mechanism by which estrogen exposure promotes breast cancer stem cell proliferation and epithelial–mesenchymal transition. Tamoxifen resistant cell lines show aberrant activation of Hh signaling, and knockdown of Hh pathway inhibited growth of tamoxifen resistant cells. As in other cancers Hh signaling is activated by the PI3K/AKT pathway in these endocrine resistant cell lines. Hh pathway activation has also been reported to mediate chemotherapy resistance in TNBC via various mechanisms including paracrine signaling to tumor micro-environment and selective proliferation of cancer stem cells. Co-activation of Hh and Wnt signaling pathways is a poor prognostic marker in TNBC. Early phase clinical trials are evaluating the combination of smoothened (SMO) inhibitors and chemotherapy in TNBC. In addition to SMO inhibitors like vismodegib and sonidegib, which are in clinical use for basal cell carcinoma, GLI1 inhibitors like GANT58 and GANT61 are in preclinical drug development and might be an effective mechanism to overcome drug resistance in breast cancer. Gene signatures predictive of Hh pathway activation could enrich for patients likely to respond to these agents.

## 1. Introduction

Breast cancer accounts for about one-third of cancer diagnoses in women. In 2019, there are predicted to be around 268,000 new cases of breast cancer in the United States and about 42,000 breast cancer related deaths [1]. About 75% of breast cancer cases are hormone receptor positive (HR+), while 15% are human epidermal growth factor receptor 2 (HER2) positive and 10% are triple negative [2]. Endocrine therapy is the major treatment modality for HR+ cancer, and the recent emergence of CDK4/6 inhibitors has extended median progression free survival (PFS) for patients with metastatic breast cancer to 24 months [3,4,5]. Although the availability of endocrine therapies has improved the outcomes of HR+ breast cancer, the development of endocrine resistance is inevitable and precludes the possibility of long-term disease control. For patients with triple negative breast cancer (TNBC), treatment options are typically restricted to cytotoxic chemotherapy [6,7]. As a result, there is a great need to develop new targets in TNBC and new treatment options to overcome endocrine resistance in HR+ breast cancer.

The hedgehog (Hh) pathway is highly conserved and is critical for normal embryogenesis [8,9]. The Hh pathway has been implicated in regulating embryonic mammary gland induction, development of ductal architecture and differentiation in lactation [10]. Dysregulation of the pathway is implicated in the development and proliferation of breast cancer [11,12]. The Hh protein family consists of Hh ligands (Sonic-SHH, Indian-IHH, and Desert-DHH) which bind cell surface transmembrane receptor Patched (PTCH). PTCH inhibits the activity of the transmembrane receptor like protein Smoothened (SMO), but when bound to ligands (SHH, IHH, DHH) it releases SMO which results in posttranslational processing of the GLI (glioma-associated oncogene homolog)–zinc-finger transcription factors. Thus far, three mammalian GLI proteins have been identified; GLI1 and GLI2 usually act as transcriptional activators, and GLI3 acts as a transcriptional repressor [13]. Hh pathway mutations like *PTCH1* are known to be oncogenic in basal cell carcinoma and have been successfully targeted with small molecule inhibitors of smoothened (SMO) like vismodegib [14]. Other mechanisms of aberrant activation like overexpression of Hh ligand, autocrine and paracrine signaling are identified in several other cancers like lung cancer, colorectal cancer, prostate cancer, breast cancer and malignant melanoma. Here, we discuss the activation of the Hh pathway in HR+ breast cancer and TNBC and the potential for therapeutic targeting.

## 2. Hedgehog Signaling in Mammary Gland Development and Cancer

**Figure 1:**
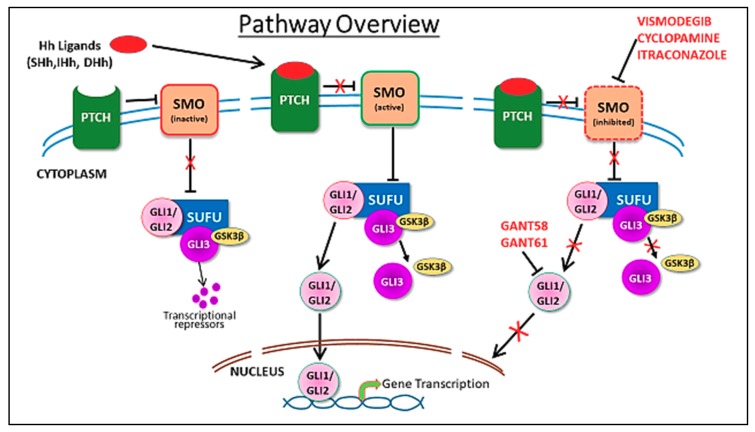
Figure depicts canonical hedgehog signaling pathway. In absence of hedgehog ligands, sonic hedgehog (SHH), Indian hedgehog (IHH) and desert hedgehog (DHH) bind to Patched (PTCH) protein, which inhibits Smoothened (SMO) and its downstream signaling events. Binding of Hh ligands to PTCH inhibits the PTCH, leading to the dis-inhibition of SMO, which then inhibits suppressor of fused (SUFU), thereby leading to release and nuclear translocation of glioma-associated oncogene (GLI)1/2 proteins and transcriptional upregulation of various GLI target genes. GLI3 is a transcriptional repressor, that when associated with SUFU in a trimolecular complex with GSK3b, undergoes further processing to generate repressor GLI. Activation of SMO leads to dissociation of SUFU/GLI3/GSK3b complex. Inhibitors of the pathway, including vismodegib, cyclopamine, itraconazole, GANT58 and GANT61 and the sites of inhibition are indicated.

The Hh pathway is a complex pathway that can be divided into canonical and non-canonical components [15]. Activation of the canonical pathway involves the release of its ligands: sonic Hedgehog, desert Hedgehog or Indian Hedgehog. These ligands bind and inhibit a transmembrane receptor, PTCH1, leading to activation of the pathway (Figure 1). In the unbound state, PTCH1 inhibits the transmembrane transducer SMO. Ligand binding to PTCH1 relieves the repression of SMO by PTCH1, resulting in translocation of SMO to the primary cilium. This initiates an intracellular signal cascade that promotes dissociation of suppressor of fused (SUFU) from GLI resulting in activation of the transcription factors. Activated GLIs in the cytoplasm then translocate to the nucleus and promote transcription of Hh target genes. There are three GLI proteins: GLI1 (a transcriptional activator), GLI3 (a transcriptional repressor) and GLI2 (acts as both a repressor and activator). In the cytoplasm, GLI proteins are degraded by the proteasome through phosphorylation by protein kinase A, casein kinase 1 and glycogen synthase kinase 3β (GSK3β). Numerous Hh target genes are described which are involved in cell cycle regulation (Cyclin D1/2) [16], proliferation (PDGFR, MYC) [17], apoptosis (BCL2) [18], angiogenesis (VEGF, ANG1/2) [19], epithelial–mesenchymal transition (MMP9, SNAIL) [20,21] and stem cell regulation or self-renewal (NANOG, SOX2) [22,23,24].

Unexpectedly, active Hh signaling is not required for normal embryonic and postnatal mouse mammary gland development [25]. For example, sonic hedgehog is not required for normal mammary gland development [26,27]. GLI1 loss does not have any visible effect on normal mammary gland development and, notably, expression of GLI3, a repressor of GLI1 signaling and, therefore, of Hh signaling, is critical at multiple stages of embryonic mammary and nipple development [28]. Moreover, GLI3 deficiency causes lack of two pairs of mammary buds in mice, suggesting that active Hh signaling may interfere with normal mammary gland development [12]. Indeed, PTCH1 haplo-insufficiency causes defects in murine mammary gland development and targeted expression of GLI1 in the mammary gland causes disruption of pregnancy induced maturation and lactation failure [29,30]. Lack of Hh target gene expression is a distinguishing feature of mammary buds from alternative embryonic epidermal development pathways, such as hair follicle development [12].

However, transgenic expression of GLI1 under the regulation of the mouse mammary tumor virus promoter (MMTV) is associated with hyperplasia, defective terminal end buds and tumor development, suggesting that the disruptive effects of Hh activation may be due to altered differentiation [31]. This is further supported by the finding that Hh signaling sustains normal mammary stem cells but is down-regulated upon their differentiation [32].

**Figure 2:**
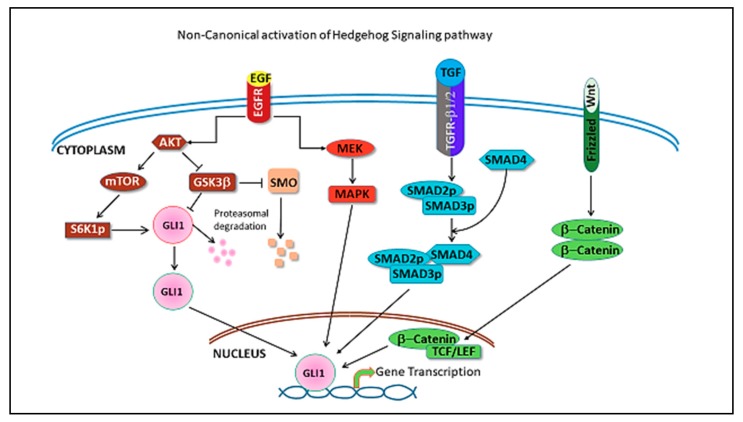
Figure depicts pathways that cross-talk with Hh pathway leading to its non-canonical activation. EGF(Epidermal growth factor) signaling pathway activates GLI mediated transcription through pAKT, as well as MAPK activation (shown in brown/red). TGFβ activation is mediated through SMAD2/3/4 complex (shown in blue) and Wnt signaling pathway activation is through b-catenin/TCF-LEF (shown in green).

In the non-canonical pathway, activation of the GLI transcription factors occurs independently of upstream components of the Hh pathway, Hh ligands, PTCH and SMO, by cross talk with other signaling cascades. MAPK, PI3K, TGFβ, Wnt, NF-κB have been described to activate the pathway (Figure 2) [15,33,34,35]. Paracrine signaling and activation of the Hh pathway in stromal cells by ligand overexpression in tumor cells leading to increased invasiveness and metastasis has been described for breast cancer and pancreatic cancer [36,37].

## 3. Hedgehog Pathway in Hormone Receptor Positive Breast Cancer

The discovery in the 1970s of tamoxifen, a selective estrogen receptor modulator, has led to a major breakthrough in the treatment of HR+ breast cancer. However, the development of resistance to endocrine therapy in HR+ breast cancer is associated with poor outcomes and remains a major challenge in the treatment of these patients. There are multiple mechanisms proposed for the development of endocrine resistance [38,39]. Our group showed that non-canonical Hh signaling is activated in tamoxifen resistant tumors [40]. Moreover, our group showed that there is cross talk between the PI3K/AKT and Hh pathway that promotes the development of tamoxifen resistance. Activation of the PI3k/AKT pathway in tamoxifen resistant cells attenuates proteasomal degradation of the Hh pathway components, GLI1 and SMO, leading to non-canonical activation of the Hh signaling pathway. We noted a consistent increase in the expression of SHH, SMO and GLI1 that correlated with the level of tamoxifen resistance in breast cancer cell lines [40]. Serial passage of tamoxifen resistant breast cancer cells in mice led to acquisition of increased aggressiveness phenotype, apparent from micro-metastasis in distant organs. This was concurrent with increased expression of SMO and GLI1 in the tumors. We also noted increased expression of vimentin, decreased expression of E-cadherin [41] (important steps in epithelial–mesenchymal transformation or EMT) and increased mammosphere formation with serial passages and a more mesenchymal phenotype. On the other hand, knockdown of Hh pathway components inhibits growth of tamoxifen resistant cells. This data demonstrates the role of Hh activation in the acquisition of aggressive phenotypes by tamoxifen resistant tumors.

In a tissue microarray of 315 patients with invasive breast cancer, high epithelial GLI1 expression was independently associated with shorter disease-free survival (*p* = 0.0375, Hazard Ratio 1.954, confidence interval (CI) = 1.03–3.671) [40]. The PI3K/AKT pathway is known to be activated in endocrine resistant breast tumors. Treatment with PI3K inhibitor resulted in a decrease in GLI1 dependent luciferase activity in endocrine-resistant, but not in estrogen-dependent cells. Vismodegib, a small molecule inhibitor of SMO, inhibited growth of tumors in tamoxifen resistant xenografts. These results provide the rationale for targeting the Hh pathway in endocrine resistant cancers. Combined targeting of the PI3K/AKT and Hh pathway might also be a rationale strategy to overcome resistance [40]. Our group also identified co-expression of GLI1 and two GLI1 targets, EGFR and SNAI1, to be associated with worse disease-free survival in HR breast cancer [42]. It is therefore important to develop biomarkers such as the GLI1 dependent gene expression signature to identify patients who are likely to respond to Hh directed therapy.

GLI1 has been shown to regulate cancer stem cells in various malignancies [19,43,44]. Sun et al. found that estrogen receptor expression positively correlated with GLI1 and cancer stem cell marker aldehyde dehydrogenase 1 (ALDH1) in human estrogen positive breast cancer cell lines (MCF-7 and HCC1438) [21]. Moreover, GLI1 expression increased in estrogen treated cells compared to controls. Knockdown of GLI1 inhibited estrogen induced sphere formation. Flow cytometry analysis found statistically significant expansion of CD44+/CD24− stem-like cells with estrogen treatment in the control cell line but not in GLI1 knockdown cell line. They also found GLI1 to mediate estrogen induced EMT in breast cancer cells. Expression of estrogen receptorpositively correlated with GLI1 and ALDH1 in tissue microarray from 100 human breast cancer samples [21]. In conclusion, they showed that estrogen promotes cancer stem cell development and EMT through GLI1. Cancer stem cells have been proposed to mediate endocrine resistance in HR+ breast cancer [45].

Hh activation might also play a role in progression from ductal carcinoma in situ to invasive ductal carcinoma. Souzaki et al. used GLI1 nuclear staining ratio (%GLI1 nuclear translocation) as a surrogate for Hh pathway activation [46]. The %GLI1 nuclear translocation correlated with invasiveness, and there was a serial increase in %GLI1 translocation from ductal carcinoma in situ to invasive ductal carcinoma. Activation of the Hh pathway in cancer cell lines increases the invasiveness and %GLI1 nuclear translocation of cancer cells, and inhibition of the pathway inhibited both [46]. As in other studies, estrogen increased GLI1 expression and %GLI1 nuclear translocation and invasiveness of HR+ breast cancer cells [46].

## 4. Hedgehog Pathway in Triple Negative Breast Cancer

Triple negative breast cancer (TNBC), characterized by the lack of estrogen, progesterone and HER2/neu receptors, is associated with the worst prognosis among breast cancer sub-types [47,48]. The Impassion130 trial revealed modestly improved PFS for patients treated with protein-bound microtubule inhibitor abraxane combined with the PD-L1 antagonist atezolizumab versus abraxane alone, establishing the role of immunotherapy for a selected population with metastatic TNBC that is PD-L1 positive [49]. The OlympiAD and EMBRACA trials demonstrated the efficacy of the poly ADP (adenosine diphosphate) ribose polymerase inhibitors olaparib and talazoparib in BRCA( breast cancer gene) deficient breast cancer [50,51]. However, cytotoxic chemotherapy remains the primary therapeutic modality. The development of effective therapies selectively attenuating the pathways involved in TNBC viability remains a priority.

Elevated expression of the Hh ligand, sonic hedgehog, in TNBC correlates with inferior overall survival [52]. In addition, basal expression levels of GLI1 and GLI2, which are downstream of Hh ligands, are higher in TNBC than in HR+ breast cancer, suggesting that this pathway may be especially relevant to this sub-type [53]. Moreover, GLI1 expression in HR negative breast cancer is predictive of a poor outcome, and GLI 1 knock out breast cancer cells demonstrate reduced viability [54].

The elevated expression of downstream components of the Hh pathway such as GLI1 and GLI2, in the absence of proportionately elevated Hh ligand expression, is suggestive of non-canonical activation. Although activation of the Hh pathway by somatic mutations is rare in breast cancer [55,56], non-canonical Hh activation through transcriptional upregulation of GLI1 is seen downstream of multiple oncogenic pathways, including the PI3K-Akt-mTOR pathway [40], K-Ras, c-Myc, Wnt-beta catenin and TGFβ [57]. Moreover, aberrant transcriptional upregulation of GLI1 is seen downstream of NF-κB in claudin low breast cancer, a sub-type of TNBC [33]. Additionally, the Forkhead Box C1 (FOXC1) transcription factor, an upstream mediator of Hh signaling via upregulation of GLI2 expression in basal-like breast cancer cells is induced by NF-κB [58,59]. Thus, multiple, apparently distinct pathways, interact with and activate the Hh pathway by direct transcriptional upregulation of GLI1, bypassing canonical Hh-PTCH1-SMO signaling. As a result, non-canonical Hh pathway activation, leading to elevated GLI1 and GLI2 expression, is the predominant mechanism of Hh pathway activation in TNBC. This could explain the sensitivity of TNBC xenografts to GLI inhibitors in preclinical models [53].

Hh pathway activation may be especially relevant to the viability of the CD44+ve/CD24−ve ALDH1 expressing breast cancer stem cell (BCSC) population. BCSCs are believed to be especially resistant to therapy due to their relative quiescence and expression of drug efflux pumps [60]. Hh pathway activation mediates the clonogenic re-growth of breast cancer cells after chemotherapy treatment, suggesting a role in maintenance of the breast cancer stem cell population [53,61]. Isoforms of p63, a member of the p53 tumor suppressor family, play a key role in stem cell regulation [62]. The ΔNp63 isoform of p63, which is the predominant isoform expressed in mammary epithelium, maintains the multipotent stem cell population in normal epithelium as well as in breast cancer; ΔNp63 drives expression of Hh ligands, PTCH1, GLI1 and GLI2 [63]. Consistent with this finding, inhibitors of the Hh pathway, GANT61 and Thiostrepton, were shown to attenuate stem cell phenotypes such as CD44+/CD24−ve cells and sphere forming capacity in TNBC cell lines [53,64]. An additional mechanism for promotion of the stem cell phenotype by Hh activation may be through the re-programming of cancer-associated fibroblasts (CAFs) towards a cancer stem cell supportive phenotype. Specifically, Hh signaling promotes activation of fibroblast growth factor 5 (FGF5) and fibrillary collagen secretion by CAFs [65,66]. These studies identify Hh signaling as a novel mediator of cancer stem cell plasticity through CAFs and an exciting new therapeutic target in TNBC.

Hh pathway activation may also promote invasiveness of breast cancer cells: GLI1 enhances the invasiveness of HR negative cells by upregulating matrix metallopeptidase 11 (MMP-11) [67]. Additionally, a novel alternatively spliced version of GLI1, namely truncated GLI1 (tGLI1) may play a critical role in breast cancer invasiveness by upregulating vascular endothelial growth factor-A and CD24 [68]. The Hh pathway may promote angiogenesis in TNBC xenografts through several mechanisms including its effects on VEGF-A [69]. VEGF receptor neuropilin-2 (NRP2) and GLI1 form an autocrine loop leading to a mutual increase in expression and Ras/MEK signaling activation that contributes to initiation and progression of TNBC [70]. In a tissue microarray on 200 TNBC patients, GLI1 overexpression correlated with VEGF receptor 2 expression [69]. Furthermore, the inflammatory cytokine osteopontin, which is a classically described target of ERα mediated transcriptional upregulation, upregulates mRNA expression of a number of genes associated with EMT and induces chemo-resistance to doxorubicin, paclitaxel and cisplatin by inducing the nuclear localization of GLI1 via the Akt-GSK3β pathway [71].

Thus, in summary, preclinical evidence suggests that the Hh pathway may play a critical role in the maintenance of the cancer stem cell phenotype, activation of cancer-associated fibroblasts, invasive behavior and angiogenesis in TNBC, and the mechanism of activation is mostly non-canonical via direct transcriptional upregulation of GLI1 and GLI2.

## 5. Hedgehog Pathway Inhibitors

The Hh pathway can be potentially inhibited through several strategies such as antibodies to Hh ligands, inhibition of SMO and inhibition of GLI [72,73]. The naturally occurring chemical cyclopamine with a high affinity for SMO was one of the earliest compounds that led to an increased understanding of the Hh pathway in cell lines and animal models [74]. However, the low potency and poor solubility of cyclopamine has limited its clinical use. The SMO inhibitors vismodegib [14] and sonidegib [75] are FDA (food and drug administration) approved for the treatment of advanced, unresectable or metastatic basal cell carcinoma. Other SMO inhibitors like saridegib [76], taladegib [77,78] and glasdegib [79] are also in clinical trials. SMO mutations causing secondary resistance to SMO inhibitors are reported in basal cell carcinoma [80]. Antifungal drug itraconazole is an SMO inhibitor with a distinct mechanism of action compared to other SMO antagonists and is reported to retain Hh inhibition in drug resistant SMO mutants [81,82].

As discussed above, GLI1 is activated through non-canonical pathways in several cancers including breast cancer. Activation of these pathways leads to direct activation of GLI proteins, downstream of SMO. Direct activation of GLI proteins is one of the mechanisms of resistance to SMO inhibitors. Targeted therapies against GLI, in combination with other agents like PI3K inhibitors or chemotherapy, might represent an effective strategy. For example, arsenic trioxide, widely used in acute promyelocytic leukemia, is also a GLI inhibitor [83,84]. GLI antagonists GANT58 and GANT61 interfere with GLI translocation to the nucleus and subsequent downstream transcription (Figure 1). GANT61 is the most efficient antagonist, but current evidence of efficacy is limited to preclinical studies [72,85]. GANT61 caused a dose dependent decrease in cell growth, increased apoptosis and decreased cancer stem cell population in TNBC cell lines. Combination of paclitaxel and GANT61 synergistically inhibited cell growth and cancer stem cell activity [53]. In a mouse xenograft injected with breast cancer cell line MCF-7, GANT61 inhibited primary tumor growth, EMT and distant metastasis [86]. In another xenograft model using TUBO cells, both vismodegib and GANT61 caused a significant decrease in tumor growth [87]. The degree and duration of response was significantly longer with GANT61 compared to SMO inhibitor vismodegib. GANT61 caused complete tumor regression in 80% of mice and these mice remained tumor free for up to 30 weeks [87]. GANT61 caused a significant reduction in cell proliferation, motility and invasion in GLI1 over expressing breast cancer cell lines [88]. These data provide strong preclinical evidence for potential clinical trials with GANT61 in breast cancer patients.

In addition to targeting components of the Hh pathway itself, inhibition of parallel pathways that increase Hh signaling, such as the PI3K-Akt-mTOR pathway is another potential strategy [40]. Combination of sonidegib and PI3K inhibitor buparlisib is being investigated in a Phase1b study in advanced solid cancers (NCT02303041) [89]. Other pathways which have been shown to enhance signal transmission downstream of, or in parallel to Hh activation, include the Wnt-beta catenin [90,91,92], MAPK [93], STAT3 [94], dual specificity tyrosine phosphorylation regulated kinase 1B (DYRK1B) [95], class I histone de-acetylation [96], polyamine metabolism [97] and glycolysis [98]. These pathways are potential targets in cancers with evidence of Hh pathway activation.

There is also evidence that Hh signaling alters molecular mechanisms and regulates macrophage polarization, linking Hh to a dysfunctional immune microenvironment in breast cancer [99]. In an immunocompetent mouse breast cancer model, treatment with vismodegib and GANT61 caused marked changes in the tumor micro-environment, characterized by an increase in cytotoxic immune cells and reduction in immune-suppressive innate and adaptive cells [99]. Macrophage depletion in combination with vismodegib led to a pro-inflammatory profile characterized by reduced population of myeloid derived suppressor cells, regulatory T cells concomitant with an increase in inflammatory macrophages, dendritic cells and cytotoxic T cells [99].

Clinical trials in HR+ breast cancer are limited to early phase basket trials on solid tumors. There have been limited trials of SMO antagonists for TNBC. In patient derived xenograft mouse models, SMO inhibitor treatment sensitized tumors to docetaxel chemotherapy [65]. This led to phase I EDALINE clinical trial that studied the combination of sonidegib combined with docetaxel for metastatic TNBC with a standard “3 + 3” design. A total of 3 of 12 (3/10 at the recommended phase 2 dose) patients with metastatic TNBC derived clinical benefit from combination therapy, with one patient experiencing a complete response (NCT02027376) [100]. There were no dose limiting toxicities. Grade 3 adverse events (AEs) included neutropenia (66.7%), increased creatine phosphokinase (33.3%) and paresthesia (33.3%) with no grade 4 AEs. An ongoing trial of vismodegib combined with standard neo-adjuvant chemotherapy, consisting of epirubicin and cyclophosphamide followed by paclitaxel, is currently recruiting patients (NCT02694224) to study the effects on pathological complete response rate in patients with localized TNBC.

## 6. Conclusions

Hh pathway activation is a poor prognostic indicator in both HR breast cancer and TNBC. This complex pathway leads to several aggressive features like EMT, development and maintenance of cancer stem cells, angiogenesis and invasiveness. Despite the growing evidence for the role of the Hh pathway in various cancers, the current Hh targeted therapies in clinical trials are limited to SMO inhibitors. This strategy is unlikely to be effective in breast cancer due to downstream, non-canonical activation of GLI by other pathways such as the PIK3/AKT, EGFR, TGF-β and NF-κB pathways. Selective and potent GLI inhibitors in combination with other targets guided by biomarkers predictive of pathway activation might represent a more effective strategy.
